# Transformation Beyond COVID-19: Accessibility in Lived Experience Involvement in Research Post Pandemic. Experience, Reflections and Future Direction From the Closing the Gap Network and OWLS Study

**DOI:** 10.3389/fpsyt.2022.872341

**Published:** 2022-04-27

**Authors:** Lauren Walker, Suzanne Crosland, Gordon Johnston, Emily J. Peckham

**Affiliations:** ^1^Mental Health and Addiction Research Group, University of York, York, United Kingdom; ^2^Independent Researcher, Clackmannan, United Kingdom

**Keywords:** patient and public involvement (PPI), co-design, COVID-19, pandemic, severe mental ill health (SMI), accessibility

## Abstract

Research literature published during the COVID-19 pandemic highlights the loss of involvement opportunities for people with lived experience during the pandemic as well as the vital role lived experience advisors play at all times, including highlighting unseen aspects of the impacts of crises such as the COVID-19 pandemic. During the pandemic, researchers from the Closing the Gap Network (CtG) at the University of York worked to expand and diversify patient and public involvement (PPI) whist working on a study exploring the impact of the pandemic and associated restrictions on those with the most severe forms of mental ill health. CtG had a strong record of patient and public involvement pre-pandemic and researchers wanted to ensure that this continued during the pandemic. This paper describes the experience of lived experience involvement during the pandemic from multiple perspectives and makes recommendations for future involvement models, accessibility and recommendations for future research.

## Introduction and Context

The “Closing the Gap” network (CtG) (a research network addressing the physical health inequalities in people with the most severe forms of mental ill health) was established in 2018 to improve the physical health of people with severe mental ill health (SMI) such as schizophrenia, schizoaffective disorder and bipolar disorder. On average people with SMI die 15–20 years earlier than those in the general population (1, 2). A significant contributor to this mortality gap is preventable physical illnesses.

## Closing the Gap Network—Patient and Public Involvement and Co-Production: Pre-Pandemic Experiences

PPI engagement in health research is an important way to strengthen links between academic researchers and people using health services including mental health services. It empowers groups and individuals who are less likely to be heard in the arena of scientific research. Consequently, done well, it can challenge the dynamics of power resulting in, amongst numerous benefits for both researchers and public contributors, the opportunity for more meaningful and relevant findings. This includes providing a kind of “peripheral vision” leading to findings that may challenge current understandings in ways which researchers alone may not see. At the time of writing, it is a responsibility for researchers to work in this way (details on PPI involvement are requested by NHS Ethics Committees during the application process) (3) and to continually strive to improve PPI engagement to ensure that research is grounded in the reality of those whose health it is intended to impact and improve.

Stigma, power differentials and institutional bias (both conscious and unconscious) can be a barrier to including and working together with experts by experience and to publishing co-produced work. In the authors' experience, this can show itself in publishing, for example, where contributors to a publication are expected to have an institutional affiliation by many journals. When experts by experience do not have this, barriers and exclusion can lead to external and internal stigma within and toward those in a position of less power.

“*Co-production involves a number of challenges including power differentials, resourcing constraints, a drive for rapid progress, lack of co-production expertise, and organizational policies. Addressing power differentials is especially vital to facilitating true co-production”*(4)

CtG has a strong record of lived experience involvement in research through the LEAP (Lived Experience Advisory Panel) who have advised and guided studies into the health and wellbeing and health inequalities experienced by those with SMI. The CtG LEAP was originally formed in 2018 when York based participants in a large-scale cohort study were sent an invitation to contact the research team if they were interested in PPI. A researcher contacted respondents and those who were interested attended a face-to-face meeting. Members of this face-to-face meeting went on to become the CtG LEAP.

During the pandemic, the research team uncovered specific experiences, such as digital exclusion. In response, researchers used their existing networks to seek and actively recruit new lived experience advisors for the LEAP with these specific experiences.

“*It is important for researchers to involve those with lived experience to keep the research real and relevant rather than pure academic research which can get lost in the essentials of the research.”*

(LEAP member 1)

“*Using people with lived experience of serious mental health problems in aspects of research can provide insight into the thoughts and experiences of people actually living with mental health issues on a day-to-day basis. I believe that this adds a human expert view, while still producing valid academic data.”*

(LEAP member 2)

LEAP involvement has meant that research output has been relevant and impactful to people whose health it sets out to improve. This cannot be done by research in isolation of lived experience, and obtaining advice and opinion from a wide range of people with differing experiences within the SMI community is a priority to the researchers.

“*Patients and the public have the right to be more than just participants in research, and their involvement can lead to better outcomes”* (5)

The involvement of lived experience advice ensures that content and delivery of research is maximized in terms of relevance and acceptability for the population. Lived experience contributors are offered payment in accordance with the former INVOLVE guidelines (6) and are acknowledged in all outputs unless anonymity is requested.

The pre-pandemic method of engagement and consultation with the LEAP was generally conducted *via* face-to-face meetings. Decision-making and work with lived-experience co-applicants/co-authors was conducted *via* a mixture of face-to-face, email, and phone communication.

Pre-pandemic work had led to a consultation, engagement and advice model with positive relationships, consistency and trust between researchers and LEAP members as well as co-design and co-production work with a smaller number of individuals. Engagement through dialogue and debate had led to high levels of involvement and positive impact on research including initial questions identified by LEAP, surveys piloted with LEAP members and discussion of data and meaning/implications from a lived experience perspective.

“*Co-production identifies, validates and utilizes service users' strengths, supports people's participation and fosters engagement between services and service users. Thus co-production very much fits within a recovery oriented framework”* (4)

“*I found it useful in many ways. Contact with other people that have a serious mental health was good but working together on a shared project was enlightening and of more benefit to me than just meeting in a self-help group*.

*Serious Mental health is important to treat and recognize and this gave me an opportunity to give something back and hopefully move treatments on”*

(LEAP member 1)

“*My views and comments were heard and acknowledged. I felt that my views were appreciated. I didn't feel pressured to do any of the work and I felt sure that if I had said that I wasn't able to do something, it would have been accepted without any questions. If people with lived experience are being asked to become involved in research, other members of the team need to understand and accept that there may be times when the expert by experience is not able to give as much input because of the influence of their mental health*.

*I found taking part was both interesting and useful. I've been involved in research / evaluation but not to such a large scale. Not only could I give my opinion, I could learn too*.

*I have many years' experience of mental health issues. Taking part in this research project has let me use that experience for a positive purpose”*.

(LEAP member 2).

## Patient and Public Involvement and Co-Production: From the Literature

“*Though (such) good examples exist, it seems that in many cases co-production and PPIE (Patient and Public Involvement and Engagement) may have unfortunately fallen by the wayside in the haste of our (COVID-19) response. Already, research Ethics Committees report that inclusion of co-production and PPIE appears to have been substantially lower in COVID-19 rapid health research, relative to “normal” research”* (7)

From the lived experience perspective of a member of the authorship team on this paper, “*There have been social media discussions of opportunities for involvement decreasing without explanation”*. Discussion was around PPI meetings not taking place regularly during the pandemic, new initiatives appearing very quickly without consultation, projects run by staff without any involvement. A general theme seen was around PPI being thought too difficult during the pandemic, staff being too busy and the perception that people would be too busy/distracted/surviving the pandemic to take part anyway.

In the experience of the authorship team, the haste and complete upturning of normal ways of life and working at the outset of the pandemic as well as the urgency to produce health research at the time may have led to the seemingly very core aspects of research work being prioritized and fast-tracked. However, co-production theory and evidence tells us that lived experience should be the core of what researchers do. Until it is embedded at the deepest level, it seems it is likely to fall by the wayside in emergency situations.

“*Our understanding and imaginations are limited by our own social experiences – politicians, civil servants, scientific experts are no different. Hearing the voices of those who are rarely listened to can radically change accepted opinions about what needs to be done. Diversity results in better decision-making”* (8)

Assumptions may also have been made in the wider health research community that continuing PPI/co-production work is too difficult and time-consuming for researchers, PPI contributors or both or that people won't want, won't have time, or can't participate during a global emergency.

“*Researchers from the Institute for Development Studies point to learning from previous pandemics. Drawing from their experience of working on the Ebola epidemic in West Africa, they argue that pandemics are not just technical problems to be solved, but are social in character. They call for more deliberation and participation to ensure that decisions reflect not only the diversity of expert opinion, but also respond to the experiential knowledge of the most vulnerable”* (9, 10)

Research by Inclusion London, which is a user-led organization of D/deaf and disabled people highlights this further disadvantage and reported in June 2020 how the impact of the COVID-19 pandemic has led to disabled people being “*abandoned, forgotten and ignored”* (11).

CtG researchers made a deliberate decision to continue, to expand PPI and co-production work, and to challenge these assumptions, a decision which has led us to important lessons about accessibility. We have learnt from those we consult and co-produce with about how to improve our work for the future and we share these lessons here.

## Patient and Public Involvement and Co-Production During the COVID-19 Pandemic—the OWLS Study

The advent of the COVID-19 pandemic has had an immense impact on all members of society. It is now known that certain sectors of society have been more severely and negatively impacted than others by the pandemic and associated restrictions (12). Those more severely impacted include, people on low incomes, BAME people and those with pre-existing health conditions.

There was a heightened awareness of the potential for working in isolation from lived-experienced advisors when the pandemic restrictions began. Researchers' work had moved to home and online and the LEAP would, in normal times, be consulted at in-person meetings. The sense of urgency and change to researchers' working lives could easily have led to a minimization of lived experience contribution to research work and the detrimental impacts on relevance that would likely have had.

LEAP members' lives had clearly also been deeply impacted by the restrictions. Working lives may have been impacted in similar ways to those of the researchers or in ways which were very different. We now understand that demographics and inequalities across society greatly influenced the impacts and changes to work and lifestyle for different individuals and communities.

In March 2020 at the dawn of the COVID-19 pandemic restrictions in the UK, researchers from CtG worked to develop the OWLS (Optimizing Well-being in Self-Isolation) study through an iterative process during daily remote video meetings. The aim of the OWLS Study was to explore the effects of the pandemic and the pandemic restrictions on people with SMI. Domains explored included physical and mental health, access to health services, loneliness and social isolation, health risk behaviors, and digital connectivity. The study consisted of surveys and qualitative interviews with people with SMI.

The OWLS study included a LEAP providing consultation and engagement input as defined by the Ladder of Co-production (13), whilst decision-making and conducting of research activities were informed and conducted by a lived experience co-applicant and co-author of this paper, co-design and co-production according to the Ladder of Co-production model (13).

The research team were fortunate to have an already established LEAP and believed that it was important and necessary to continue involvement but realized that it would need to look different practically speaking.

Lived experience advisors were contacted remotely (by video call, phone, or post) regarding the OWLS study and consultation and discussion by a variety of methods was conducted to suit individual preference. A mix of online meetings, telephone calls with researchers and posting hard copies of information to be discussed over the phone to those who were experiencing digital exclusion meant that those with lived experience could highlight the issues they thought were important and likely to have the most impact as a result of the pandemic. There were a great number of similarities to the concerns of the general population, but because of the already established health related inequalities faced by people with SMI, there were specific concerns, such as access to health services, worsening physical health and increase in health risk behaviors learnt through previous LEAP engagement, unique to the population and the ways in which people with SMI may be affected and further disadvantaged.

The case for good involvement remained strong during the pandemic. This was reflected in the OWLS 2 and 3 surveys which progressed and changed in their content as part of in iterative process in response to our findings and PPI work. Indeed, it could be argued that the need to ensure the voice of lived experience was strong in research became even more important.

“*Coproduction under the pressures of the COVID-19 pandemic is challenging and risks being seen as an added extra rather than as fundamental to a successful, sustainable response'* and ‘*It is crucial to understand, for instance, the additional needs of particular groups, and the lived experiences of difficulties caused by government restrictions”* (14)

Barriers to engagement for those with lived experience of SMI took both new and familiar forms. Individual circumstances meant that ease and accessibility of involvement might have increased for some. Changes to routines through loss of voluntary, social, educational and work activities meant that some people had more time available to be involved in research. For some more isolated people, research involvement created social interaction missing due to pandemic restrictions and possibly increased connectedness in comparison to pre-pandemic times. For others, time was limited due to increased family or caring commitments such as home-schooling children. Lack of digital connectivity, which is higher in the SMI population than the general population (15) led to difficulty with continuing commitments for some, collaborative and creative working between researchers and lived experience advisors aimed to create a range of solutions such as phone, video platform and postal involvement.

The OWLS team are aware of the impacts of intersectionality and multiple discrimination on the experiences of people using mental health services in the context of the pandemic and more widely. People from BAME backgrounds are over represented in mental healthcare services and the layered effects of COVID-19 and the restrictions may have impacted on BAME people with SMI in ways that are not heard when the voices of BAME experts by experiences are not amplified enough. As a research team, we aim to work to include BAME voices.

For those who experienced digital exclusion and therefore generally fed into research on a one-to-one rather than group basis with a researcher, individual engagement meant missing out on group discussion and synthesis of ideas through debate. The authors and research team are aware of the possible shift in power dynamics due to one to one engagement with a researcher in contrast to a group dynamic with peers and the impact this may have on feedback provided.

In the initial development of our OWLS Study (March–June 2020) views of the lived experience panel (LEAP) were invited to determine the concerns of people with SMI during the pandemic and to work together to influence key domains for the research. The LEAP specifically influenced the addition of questions about use of the internet and digital devices to the quantitative study which was followed by qualitative interviews about digital usage. Through consultation, the original study title chosen by the research team (Optimizing Wellbeing during Lockdown) was changed to be sensitive to the experiences of those people with SMI who may have experienced involuntary hospitalization and for whom the term “lockdown” may have had unpleasant and frightening connotations. This change of term shows the importance of lived experience input to create a study that does not cause undue upset or distress to those it aims to research.

Discussion between the research team and the LEAP highlighted how uncertainty about the future impacted on how people viewed their future mental health. This led the research team to formulate a question about input from mental health services in the future.

The OWLS questionnaire was piloted with members of the LEAP to assess suitability of language, engagement and estimated length of completion. OWLS qualitative interview schedules were also piloted with the LEAP, including an individual with lived expertise of digital exclusion. Feedback on relevance and length was invited.

LEAP members contributed their experience and suggestions to feed into the design of a diverse recruitment strategy, which included phone, online, and postal options for completing the questionnaire.

Ideas about dissemination methods were shared with the LEAP, which led to the development of social media outputs and info-graphic, which was posted to participants accompanied by a thank you card with flower seeds that could be planted. Additionally, at the point of dissemination, our peer researcher / co-applicant contributed to and/or commented on all OWLS research outputs.

Consultation continued as the pandemic progressed to determine additional concerns as they arose. A researcher and clinician on the team commented that “*Without PPI input on this particular study, we wouldn't have known how involvement from secondary mental health services had changed for people during the early part of the pandemic. This knowledge enabled us to ask more pertinent questions regarding provision of mental health care services and the level of satisfaction around this”*. Based on lived experience insights, various questionnaire domains were agreed upon and either new questions devised or pre-existing questions used. Existing measures provided the important advantage of the ability to map findings against the general population. The questionnaire was then piloted with members of our PPI group to assess suitability of language, engagement, and estimated length of completion. OWLS qualitative interview schedules were piloted with lived experience advisors, inviting feedback on relevance and length. Lived experience interpretation and feedback on results facilitated a deeper understanding of findings and provided suggestions for next steps.

Lived experience involvement remained at a level consistent with pre-pandemic and new members were also added to the LEAP with specific experiences relevant to pandemic research such as digital exclusion and from a wider geographical area. These new voices and experiences will continue to influence the development of research work into the future.

## Moving forward: Conclusions, Recommendations and Limitations

Good involvement is always important to good research. Cummings et al. (16) suggest using FINER criteria to create or evaluate a research question. According to this set of criteria, a good research question is: (F)easible, (I)nteresting, (N)ovel, (E)thical, and (R)elevant. Good involvement specifically creates research which is interesting (presents a different perspective of the problem) ethical and relevant, points which have been expanded upon in connection to lived experience input earlier in this paper.

The pandemic has shown that whilst face-to-face involvement has many benefits, it is still possible to have good involvement under extreme circumstances if the will is there to address barriers.

For some people involved in PPI and co-production, going back to face-to-face will be their preferred option for involvement, while for others remote engagement may suit them better. For those with caring responsibilities for example, attending in-person meetings can prove challenging and so there may be a preference for online involvement.

If we are to involve as many people as possible, the challenge for researchers is to facilitate a blended or hybrid series of solutions, described by a lived experience author as “*a varied menu for involvement*.” This will maximize the numbers who can be involved, increase accessibility and therefore potentially increase the impact and relevance of the research produced.

Acknowledgment and understanding of the many and varied barriers that people with lived experience face in becoming involved is required, including time, money, digital exclusion, illness experiences and medication side effects, combined with the advantages of offering blended involvement in order to allow individuals to become as involved as they want to in a manner that suits them and their circumstances best.

The authors' experience suggests a blended involvement model for the future, which offers options for contribution, keeping what worked well during the pandemic period and merging this with successful and valued aspects of the pre-pandemic approach.

Suggestions for a possible blended involvement process from a lived experience perspective include meetings with a mix of people attending in person and online. Matters to be addressed in moving such ideas forward include the equipment and planning required, support for individuals involved in PPI work and what form that might take, creating dialogue and future direction in terms of involvement, potentially including messaging and online forums and sending questions in advance to allow written input to be added to discussions.

Limitations of this work include the fact that the model leaned toward one of involvement rather than co-production. Learnings for the future might include seeking lived experience input into the need for capacity building and infrastructure to embed involvement at the core of what researchers do so that it is less likely to fall by the wayside in any future emergency situations.

The authors would suggest that there is research work to be done looking at barriers and facilitators to involvement leading to co-designing an accessible involvement strategy. Additionally, the authors' see opportunities for creative, co-produced qualitative work designing the future of embedded, accessible, meaningful involvement, which we will know to be at the core of our work. Focus on ensuring we involve people who represent the community we serve in terms of demographics is a priority and we hope that our experience provides a stepping stone to ensuring that lived experience involvement in mental health research, broader health research and in the research world more widely, will not be something which can be side-lined during future emergency situations.

## Data Availability Statement

The original contributions presented in the study are included in the article/supplementary material, further inquiries can be directed to the corresponding authors.

## Author Contributions

LW, SC, and GJ wrote the manuscript. GJ provided guidance from a lived experience perspective. SC collected qualitative data. EP read, commented on the manuscript, and provided senior and academic guidance. All authors contributed to the conceptualization of the paper, manuscript revision, read, and approved the submitted version.

## Funding

This study was supported by the Medical Research Council (Grant No. MR/V028529) and links with the Closing the Gap cohort, which was part-funded by the Wellcome Trust (reference 204829) through the Center for Future Health at the University of York, UK Research and Innovation (reference ES/S004459/1), and the NIHR Yorkshire and Humber Applied Research Collaboration.

## Conflict of Interest

The authors declare that the research was conducted in the absence of any commercial or financial relationships that could be construed as a potential conflict of interest.

## Publisher's Note

All claims expressed in this article are solely those of the authors and do not necessarily represent those of their affiliated organizations, or those of the publisher, the editors and the reviewers. Any product that may be evaluated in this article, or claim that may be made by its manufacturer, is not guaranteed or endorsed by the publisher.
